# Continuous applications of biochar to rice: Effects on nitrogen uptake and utilization

**DOI:** 10.1038/s41598-018-29877-7

**Published:** 2018-07-30

**Authors:** Min Huang, Long Fan, Jiana Chen, Ligeng Jiang, Yingbin Zou

**Affiliations:** 1 Southern Regional Collaborative Innovation Center for Grain and Oil Crops (CICGO), Hunan Agricultural University, Changsha, 410128 China; 2 Key Laboratory of Crop Cultivation and Farming System, Guangxi University, Nanning, 530004 China

## Abstract

Improving soil quality is critical for increasing rice yield, and biochar could be a beneficial soil amendment for high yield. This study was conducted to determine the effects of continuous (repeated seasonal) applications of biochar on nitrogen (N) uptake and utilization in rice. A fixed field experiment was done in Yongan Town, Hunan Province, China, in six continuous seasons (the early and late rice-growing seasons from 2015 to 2017). Results showed that biochar application did not significantly affect soil N uptake in the first four seasons. The effect of biochar application on fertilizer N uptake was not significant in three of the first four seasons. In the fifth and sixth seasons, biochar application resulted in 14–26% increases in soil N uptake but 19–26% decreases in fertilizer N uptake. Soil N availability did not explain the increased soil N uptake with biochar application. The decreased fertilizer N uptake with biochar application was attributed to both decreased fertilizer N availability and increased N loss through ammonia volatilization. As a consequence of a compensation between the increased soil N uptake and the decreased fertilizer N uptake, the effect of biochar application on total N uptake was not significant in the fifth and sixth seasons. However, biochar application led to 7–11% increases in internal N use efficiency in the fifth and sixth seasons and 6% increase in grain yield in the sixth season. Our study suggests that the effects of repeated seasonal applications of biochar on N uptake and utilization in rice depend on the duration of biochar application. Longer continuous applications of biochar can increase internal N use efficiency and grain yield in rice with insignificant change in total N uptake.

## Introduction

Rice is the staple food for more than half of the world’s population^1^. Global rice production must be increased by about 1% annually to meet the growing demand for food resulting from population growth and economic development^2^, and greater yield and sustainable intensification are required for this increase^3,4^. It is also important to minimize the dependence on external inputs (e.g. chemical fertilizers) in crop production to reduce adverse environmental impacts, such as increased greenhouse gas emissions^5^, soil acidification^6^, surface water eutrophication^7^, and biodiversity loss^8^. To enhance rice yield with fewer chemical fertilizer inputs, improvement in soil quality is critical^9,10^. An important step towards improving soil quality is to facilitate recycling of organic fertilizers including crop residues^11^.

Biochar is a carbon-rich solid material produced by pyrolyzing biomass (e.g. crop residues) in an oxygen-limited environment^12^. Biochar application to soils is considered a means of abating climate change by sequestering carbon (C)^13^. Moreover, because biochar application has the potential to stimulate crop growth by improving soil quality including enhancing water storage, improving nutrient supply, increasing beneficial microbial activity, and suppressing soil-borne disease, it has been postulated that biochar could be a beneficial soil amendment for crop production^14–17^.

Nitrogen (N) is usually the most yield-limiting nutrient in rice production^18^. Because biochar contains some N, its application could increase total N content in the soil^19^. However, there have been reports that biochar application may decrease N availability by its direct adsorption effect and/or indirect microbial immobilization effect^20–22^. On the other hand, N uptake, especially fertilizer N uptake, in rice plants is largely influenced by the magnitude of N loss^18,23^. It has been documented that biochar application can reduce nitrous oxide emission and N leaching from rice fields^24,25^. However, because biochar is typically alkaline^26^, its application can increase soil pH and may subsequently increase ammonia (NH_3_) volatilization from rice soils^27–29^. These facts indicate that biochar application may lead to changes in N uptake and utilization in rice. This was confirmed in previous studies of other crops such as wheat and maize^30,31^ and also in a one-season rice pot experiment by Huang *et al*.^32^, who observed that biochar application increased fertilizer N uptake in rice. However, it is still not clear how N uptake and utilization in rice will change in response to continuous applications of biochar under field conditions. Such information would be useful in guiding the use of biochar in rice production.

In the present study, N uptake, internal N use efficiency, and grain yield were compared between rice grown with and without biochar application in a field experiment carried out in six continuous seasons. The main objective of this study was to determine the effects of continuous applications of biochar on N uptake and utilization in rice.

## Results

The ANOVA showed that there were no statistically significant interaction effects between biochar and N rates on fertilizer and total N uptake, internal N use efficiency, and grain yield in all six seasons (Supplementary Table S1) as well as for total NH_3_ volatilization, soil ^15^N abundance, ^15^N uptake, and soil pH, total N content, available N content and invertase activity in the late season in 2017 (Supplementary Table S2). Therefore, means of biochar and N rates are presented for these parameters.

### Soil, fertilizer and total N uptake

**Figure 1:**
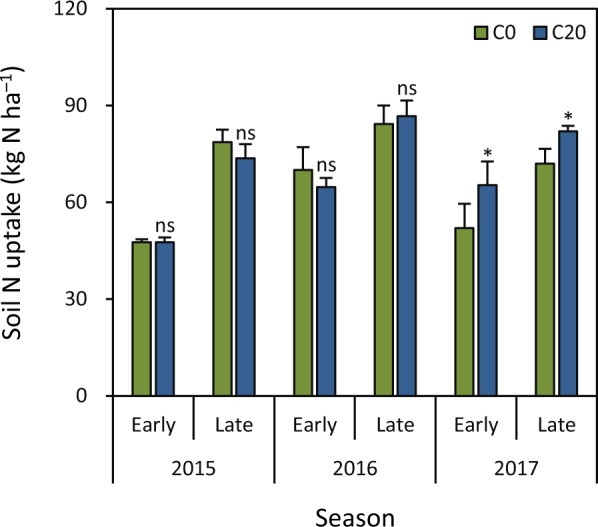
Effect of biochar rate on soil N uptake in rice grown in the early and late seasons from 2015 to 2017. C0 and C20 represent 0 and 20 t biochar ha^−1^, respectively. Error bars represent SE (*n* = 3). ns and * denote non-significant and significant differences at the 0.05 probability level, respectively.

There was no significant difference in soil N uptake between C20 (20 t biochar ha^−1^) and C0 (0 t biochar ha^−1^) in the early and late seasons in 2015 and 2016 (Fig. 1). Soil N uptake was higher under C20 than under C0 by 26% and 14%, respectively, in the early and late seasons in 2017.

**Figure 2:**
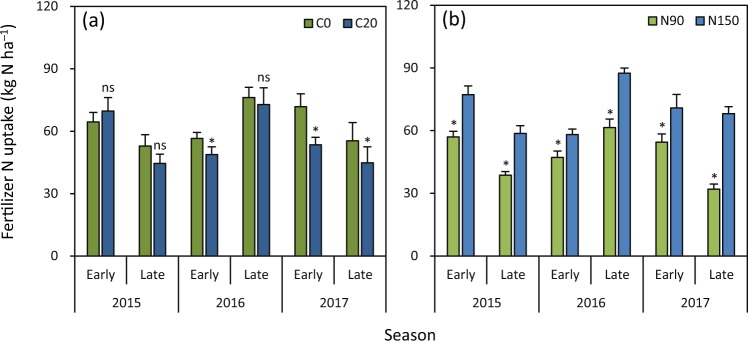
Effects of biochar rate (**a**) and N rate (**b**) on fertilizer N uptake in rice grown in the early and late seasons from 2015 to 2017. C0 and C20 represent 0 and 20 t biochar ha^−1^, respectively. N90 and N150 represent 90 and 150 kg N ha^−1^, respectively. Error bars represent SE (*n* = 6). ns and * denote non-significant and significant differences between treatments in each season at the 0.05 probability level, respectively.

The difference in fertilizer N uptake was not significant between C20 and C0 in the early and late seasons in 2015 and in the late season in 2016 (Fig. 2a). C20 had lower fertilizer N uptake than C0 in the early season in 2016 and in the early and late seasons in 2017 by 14%, 26% and 19%, respectively. Fertilizer N uptake was significantly lower (19–53%) under N90 (90 kg N ha^−1^) than under N150 (150 kg N ha^−1^) in all six seasons (Fig. 2b).

**Figure 3:**
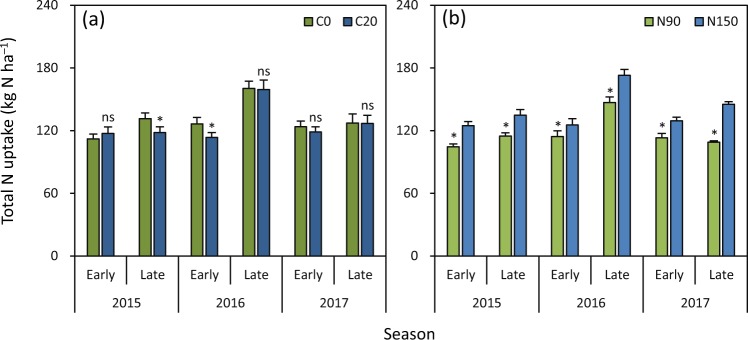
Effects of biochar rate (**a**) and N rate (**b**) on total N uptake in rice grown in the early and late seasons from 2015 to 2017. C0 and C20 represent 0 and 20 t biochar ha^−1^, respectively. N90 and N150 represent 90 and 150 kg N ha^−1^, respectively. Error bars represent SE (*n* = 6). ns and * denote non-significant and significant differences between treatments in each season at the 0.05 probability level, respectively.

There was no significant difference in total N uptake between C20 and C0 in the early season in 2015, in the late season in 2016, and in the early and late seasons in 2017 (Fig. 3a). Total N uptake was 10% lower under C20 than under C0 in the late season in 2015 and in the early season in 2016. N90 had significantly lower (9–25%) total N uptake than N150 in all six seasons (Fig. 3b).

### Internal N use efficiency

**Figure 4:**
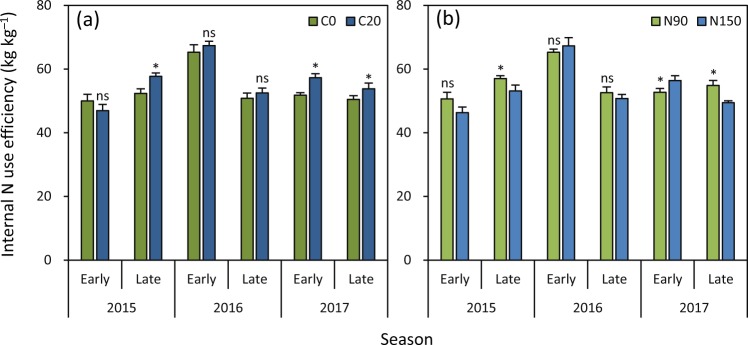
Effects of biochar rate (**a**) and N rate (**b**) on internal N use efficiency in rice grown in the early and late seasons from 2015 to 2017. C0 and C20 represent 0 and 20 t biochar ha^−1^, respectively. N90 and N150 represent 90 and 150 kg N ha^−1^, respectively. Error bars represent SE (*n* = 6). ns and * denote non-significant and significant differences between treatments in each season at the 0.05 probability level, respectively.

The difference in internal N use efficiency was not significant between C20 and C0 in the early season in 2015 and in the early and late seasons in 2016 (Fig. 4a). C20 had higher internal N use efficiency than C0 in the late season in 2015 and in the early and late seasons in 2017 by 10%, 11% and 7%, respectively. There was no significant difference in internal N use efficiency between N90 and N150 in the early season in 2015 and in the early and late seasons in 2016 (Fig. 4b). N90 had 7% and 11% higher internal N use efficiency than N150 in the late season in 2015 and 2017, respectively. Internal N use efficiency was 6% lower under N90 than under N150 in the early season in 2017.

### Grain yield

**Figure 5:**
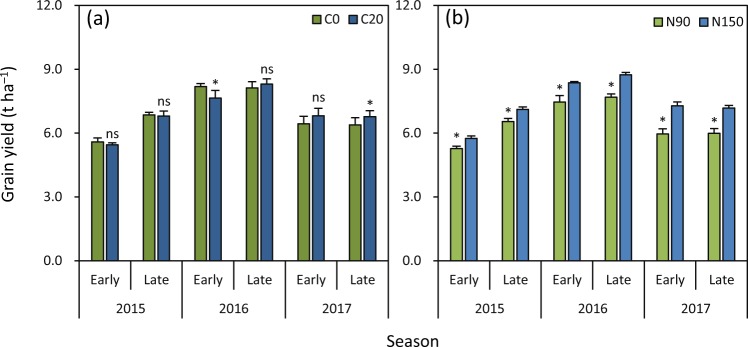
Effects of biochar rate (**a**) and N rate (**b**) on grain yield in rice grown in the early and late seasons from 2015 to 2017. C0 and C20 represent 0 and 20 t biochar ha^−1^, respectively. N90 and N150 represent 90 and 150 kg N ha^−1^, respectively. Error bars represent SE (*n* = 6). ns and * denote non-significant and significant differences between treatments in each season at the 0.05 probability level, respectively.

There was no significant difference in grain yield between C20 and C0 in the early and late seasons in 2015, in the late season in 2016, and in the early season in 2017 (Fig. 5a). Grain yield was 7% lower under C20 than under C0 in the early season in 2016. C20 produced 6% higher grain yield than C0 in the late season in 2017. Grain yield was significantly lower (8–18%) under N90 than under N150 in all six seasons (Fig. 5b).

### Total NH_3_ volatilization, soil ^15^N abundance and ^15^N uptake

**Figure 6:**
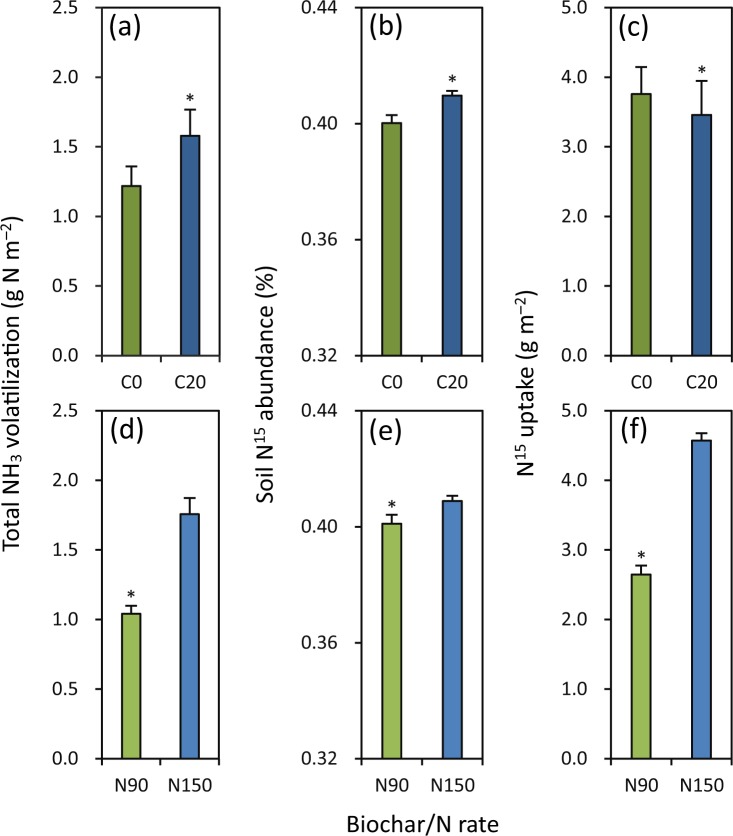
Effects of biochar rate (**a**–**c**) and N rate (**d**–**f**) on total NH_3_ volatilization (**a** and **d**), soil ^15^N abundance (**b** and **e**) and ^15^N recovery efficiency (**c** and **f**) in a ^15^N tracing micro-plot experiment in the late rice-growing season in 2017. C0 and C20 represent 0 and 20 t biochar ha^−1^, respectively. N90 and N150 represent 90 and 150 kg N ha^−1^, respectively. Error bars represent SE (*n* = 6). * denotes significant difference between treatments at the 0.05 probability level.

In a ^15^N tracing micro-plot experiment in the late season in 2017, C20 had 30% higher total NH_3_ volatilization than C0 (Fig. 6a). Soil ^15^N abundance was 2% higher under C20 than under C0 (Fig. 6b). C20 had 8% lower ^15^N uptake than C0 (Fig. 6c). Total NH_3_ volatilization was 41% lower under N90 than under N150 (Fig. 6d). N90 had 2% lower soil ^15^N abundance than N150 (Fig. 6e). ^15^N uptake under N90 was 42% lower than that under N150 (Fig. 6f).

### Soil pH, N content and invertase activity

**Figure 7:**
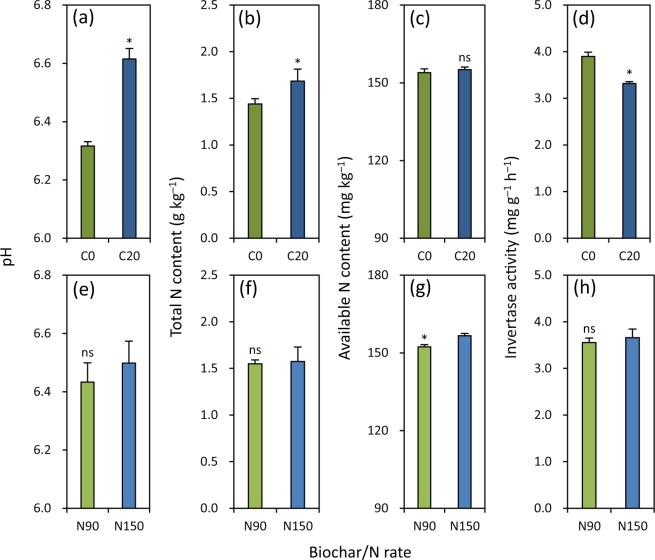
Effects of biochar rate (**a**–**d**) and N rate (**e**–**h**) on soil pH (**a** and **e**), total N content (**b** and **f**), available N content (**c** and **g**) and invertase activity (**d** and **h**) at maturity in the late rice-growing season in 2017. C0 and C20 represent 0 and 20 t biochar ha^−1^, respectively. N90 and N150 represent 90 and 150 kg N ha^−1^, respectively. Error bars represent SE (*n* = 6). ns and * denote non-significant and significant differences between treatments in each season at the 0.05 probability level, respectively.

At maturity in the late season in 2017, C20 had 5% higher soil pH than C0 (Fig. 7a). Soil total N content was 17% higher under C20 than under C0 (Fig. 7b). There was no significant difference in soil available N content between C20 and C0 (Fig. 7c). C20 had 15% lower soil invertase activity than C0 (Fig. 7d). The differences in soil pH, total N content and invertase activity were not significant between N90 and N150 (Fig. 7e,f and h). Soil available N content was 3% lower under N90 than under N150 (Fig. 7g).

## Discussion

Prior to the present study, limited information was available on the effect of continuous applications of biochar on N uptake in rice^19^. This study showed that biochar application had no significant effect on soil N uptake in the first four seasons. Fertilizer N uptake was not significantly affected by biochar application in three of the first four seasons. In the fifth and sixth seasons, biochar application significantly increased soil N uptake, but the effect of biochar application on total N uptake was not significant because it was compensated for by significantly decreased fertilizer N uptake. These facts indicate that the effects of repeated seasonal applications of biochar on soil and fertilizer N uptake depend on the duration of biochar application. Thing finding enriches our understanding of the effect of biochar application on N uptake in rice.

Soil N uptake is determined by soil N availability and root system activity^33,34^. In this study, biochar application significantly increased soil total N content at the end of the experiment. The increase in soil total N content with biochar application was partly due to the biochar containing a certain amount of N. Moreover, the ^15^N tracing micro-plot experiment indicates that increased fertilizer N retention (soil ^15^N abundance) in the soil was also partially responsible for the increased soil total N content induced by biochar application. This finding is consistent with previous reports that fertilizer N retention in the soil can be increased by biochar application through its direct adsorption effect and/or indirect microbial immobilization effect^20–22^. Furthermore, in this study, the increase in soil total N content with application of biochar did not result in increased soil available N content. This was partly because the N contained in biochar existed in organic forms and most fertilizer N retained in the soil was converted to organic forms, neither of which were readily available for plant uptake^35^. On the other hand, soil enzymes are known to be involved in nutrient cycling, and as such, their activities can be used as potential indicators of nutrient cycling processes^36^. Huang *et al*.^37^ observed a positive relationship between invertase activity and available N content in rice soils. Therefore, in this study, the insignificant effect of biochar application on soil available N content was also attributed to a trade-off between increased soil total N content and decreased soil invertase activity. These facts indicate that soil N availability was not responsible for the significantly positive effect of longer continuous applications of biochar on soil N uptake. Therefore, the increased soil N uptake with longer continuous applications of biochar application should be attributed to improved root traits. In this regard, it has been well documented that biochar application benefits root morphological development^38^. However, further investigations are required to confirm the effects of longer continuous applications of biochar on root morphological and physiological traits in rice.

Fertilizer N uptake by rice plants is closely related to fertilizer N availability and loss^18,23^. In this study, as mentioned above, fertilizer N retention in the soil was increased by biochar application in the sixth season. This indicates that longer continuous applications of biochar resulted in decreased availability of fertilizer N. On the other hand, N loss through NH_3_ volatilization increased with application of biochar in the sixth season. This increased NH_3_ volatilization was attributed to an increase in soil pH, which is generally positively correlated with NH_3_ volatilization^39^. Similar results have also been reported in short-term experiments in previous studies^27–29^. These facts indicate that the significantly negative effect of longer continuous applications of biochar on fertilizer N uptake in this study was attributed to both decreased fertilizer N availability and increased fertilizer N loss.

Biochar application had a significantly positive effect on internal N use efficiency in the fifth and sixth seasons. In general, internal N use efficiency increases with decreasing total N uptake in rice^40^. However, in this study, the significantly positive effect of biochar application on internal N use efficiency was achieved with unchanged total N uptake in the sixth season. As a consequence, grain yield was significantly increased by biochar application in this season. These results indicate that higher grain yield can be obtained after longer continuous applications of biochar by increasing internal N use efficiency. There is evidence that increasing harvest index is one possible way to achieve high internal N use efficiency in rice^41^. However, this was not the case in the present study because harvest index was significantly decreased with biochar application (data not shown). In another approach, the internal N use efficiency can be increased by increasing photosynthetic N use efficiency^42^. Consistently, we observed that biochar addition resulted in significantly higher radiation use efficiency during the post-heading period in rice plants in another study (data not shown). These facts highlight the need for a fundamental understanding of the effects of biochar application on physiological processes governing internal N use efficiency in rice.

Reducing N rate from 150 to 90 kg ha^−1^ resulted in reduction in grain yield in all six seasons. The reduced grain yield was mainly attributed to decreased total N uptake, which was caused by both decreased fertilizer N uptake and decreased soil available N content. The result of this study is not consistent with that reported by Huang *et al*.^43^, who observed that grain yield was not significantly affected by reducing N rate from 150 to 90 kg ha^−1^ in a single-season rice system. This difference in response of grain yield to reducing N was due to the different proportions of fertilizer N uptake in total N uptake. In this study, fertilizer N uptake accounted for 46% of total N uptake across six seasons (Figs 2b and 3b), while it accounted for only 18% in Huang *et al*.^43^. In addition, the results of this study also suggest that biochar application may not reduce the dependence on N fertilizer in rice.

## Conclusions

The effects of continuous applications of biochar on N uptake and utilization in rice depend on the duration of biochar application. Longer (six-season) continuous applications of biochar are able to improve grain yield in rice by increasing internal N use efficiency with unchanged total N uptake. However, the dependence on N fertilizer in rice may not be reduced by biochar application.

## Methods

### Site and soil

A fixed field experiment was conducted in Yongan Town (28°09′N, 113°37′E, 43 m asl), Hunan Province, China, in the early and late rice-growing seasons from 2015 to 2017. The experimental site has a moist subtropical monsoon climate with an annual average temperature of 17.5 °C, an annual average sunshine duration of 1595 h, and an annual average rainfall of 1551 mm.

The soil of the experiment field was a Fluvisol (FAO taxonomy) with clay texture and the following properties: pH = 6.30, organic C = 24.7 g kg^−1^, available N = 207 mg kg^−1^, available P = 7.11 mg kg^−1^, and available K = 81.1 mg kg^−1^. The soil tests were based on samples taken from the 0–20 cm layer before the experiment was started in the early rice-growing season in 2015. The pH was measured using a digital pH meter (PHS-3C, Shanghai Precision & Scientific Instrument Inc., Shanghai, China), organic C was determined using the potassium dichromate method, available N using the diffusion method, available P using the double acid method, and available K using a flame photometer (FP640, Shanghai Precision & Scientific Instrument Inc., Shanghai, China)^44^.

### Plant and treatments

In each season, an inbred rice cultivar Zhongzao 39 was used in the experiment. This cultivar has been widely grown by rice farmers in the double-season rice cropping regions in southern China. Treatments were two biochar rates, 0 (C0) and 20 t ha^−1^ (C20), with three N rates, 0 (CK), 90 (N90), and 150 kg N ha^−1^ (N150). The biochar and N were applied in each season. The tested biochar (pH 10.4, 512 g kg^−1^ total C, and 7.8 g kg^−1^ total N) was produced from rice husks by pyrolysis in a fluidized bed reactor at 600 °C for 3 h. Urea was used as the N fertilizer. Treatments were arranged in a split-plot design with N rates as main plots and biochar rates as subplots. The experiment was replicated three times and subplot size was 20 m^2^.

In the late season in 2017, a micro-plot was established in each subplot with application of N fertilizer by inserting a PVC cylinder (40 cm in diameter and 40 cm in height) into the soil at a depth of approximately 20 cm to reach the plough pan. Except for the N fertilizer using ^15^N-labeled urea (5.18% isotopic abundance, produced by the Shanghai Institute of Chemical Industry, China), the micro-plots were managed in the same way as the main subplot.

Pre-germinated seeds were sown in a seedbed. Twenty-five and 15-day-old seedlings were transplanted on 22 April and 21 July in the early and late seasons, respectively. Transplanting was done at a hill spacing of 20 cm × 16.7 cm with three seedlings per hill. Biochar was applied as basal fertilizer (1 day before transplanting). N was applied in three splits: 50% as basal fertilizer, 30% at early-tillering (7 days after transplanting), and 20% at panicle initiation. Phosphorus (75 kg P_2_O_5_ ha^−1^ as basal fertilizer) and potassium (75 kg K_2_O ha^−1^ as basal fertilizer, and 75 kg K_2_O ha^−1^ at panicle initiation) were applied in all subplots. The experimental field was kept flooded from transplanting until 7 days before maturity, when the field was drained. Insects and diseases were intensively controlled by chemicals to avoid yield loss.

### Sampling and measurements

Ten hills were sampled diagonally from a 5 m^2^ harvest area for each subplot at maturity in each season. Plants were hand-threshed and filled spikelets were separated from unfilled spikelets by submerging them in tap water. Dry weights of straw, rachis, and filled and unfilled spikelets were determined after oven drying at 70 °C to a constant weight. The dried samples were ground and sieved (100 mesh). About 0.5 g of the sieved sample was digested with H_2_SO_4_-H_2_O_2_, and the N content was measured using a segmented flow analyzer (Skalar SAN Plus, Skalar Inc., Breda, The Netherlands). N uptake was calculated by multiplying the dry weight by the N content. Grain yield was determined from a 5 m^2^ area in each subplot and adjusted to the standard moisture content of 0.14 g H_2_O g^−1^.

Total N uptake was defined as the N uptake in the subplots receiving N fertilizer (N90 and N150). Soil N uptake was defined as the N uptake in the subplots receiving no N fertilizer (CK). Fertilizer N uptake was calculated by subtracting the soil N uptake from the total N uptake. Internal N use efficiency was calculated by dividing the grain yield by the total N uptake.

In the late season in 2017, daily NH_3_ volatilization was measured in the micro-plots according to the procedures described by Huang *et al*.^29^. Total NH_3_ volatilization was calculated as the sum of the daily NH_3_ volatilization. At maturity, soils and plants were sampled from each micro-plot to determine soil ^15^N abundance and ^15^N uptake according to Huang *et al*.^32^. After harvesting the rice, soil samples were taken from the upper 20 cm layer of each subplot to determine pH, total and available N content, and invertase activity. The pH and available N content were determined according to the methods mentioned above. The total N content was measured using the semi-micro Kjeldahl method^44^. The invertase activity was determined using sucrose as a substrate and after incubation at 37 °C for 24 h, the glucose produced was measured using a colorimetric method^37^.

### Statistical analysis

Data were analyzed with analysis of variance (ANOVA) in Statistix 8.0 (Analytical software, Tallahassee, FL, USA). The statistical significance was set at the 0.05 probability level.

### Data availability

All data generated or analysed during this study are included in the article (and its Supplementary Information file).

## Electronic supplementary material

Table S1 and S2
