# Supplementary material for: Continuous applications of biochar to rice: Effects on nitrogen uptake and utilization
Source: Sci Rep. 2018 Jul 30;8:11461. doi: 10.1038/s41598-018-29877-7 (PMC6065394; doi:10.1038/s41598-018-29877-7)
Supplement: Supplementary file 1 — Table S1 and S2 [file 41598_2018_29877_MOESM1_ESM.docx]

**Continuous applications of biochar to rice: Effects on nitrogen uptake and utilization**

Min Huang^1, *, +^, Long Fan^1, +^, Jiana Chen^1^, Ligeng Jiang^1, 2^, Yingbin Zou^1^

^1^Southern Regional Collaborative Innovation Center for Grain and Oil Crops (CICGO), Hunan Agricultural University, Changsha 410128, China

^2^Key Laboratory of Crop Cultivation and Farming System, Guangxi University, Nanning 530004, China

^*^corresponding. mhuang@hunau.edu.cn

^+^these authors contributed equally to this work.

**Table S1** Analysis of variance for the effects of biochar and N rates on fertilizer and total N uptake, internal N use efficiency, and grain yield in rice grown in the early and late seasons from 2015 to 2017

| Biochar rate† | N rate‡ | 2015 | |  | 2016 | |  | 2017 | |
| --- | --- | --- | --- | --- | --- | --- | --- | --- | --- |
|  |  | Early | Late |  | Early | Late |  | Early | Late |
| Fertilizer N uptake (kg ha^–1^) | | | | | | | | | |
| C0 | N90 | 56.3 | 41.0 |  | 52.0 | 67.7 |  | 60.3 | 36.0 |
|  | N150 | 72.7 | 64.7 |  | 61.0 | 84.7 |  | 83.3 | 74.7 |
| C20 | N90 | 57.7 | 36.3 |  | 42.3 | 55.3 |  | 48.7 | 28.0 |
|  | N150 | 81.7 | 52.7 |  | 55.3 | 90.3 |  | 58.3 | 61.7 |
| Analysis of variance | |  |  |  |  |  |  |  |  |
| Biochar rate | | ns | ns |  | * | ns |  | * | * |
| N rate | | * | * |  | * | * |  | * | * |
| Biochar rate × N rate | | ns | ns |  | ns | ns |  | ns | ns |
| Total N uptake (kg ha^–1^) | | | | | | | | | |
| C0 | N90 | 104 | 120 |  | 122 | 152 |  | 112 | 108 |
|  | N150 | 120 | 143 |  | 131 | 169 |  | 135 | 147 |
| C20 | N90 | 105 | 110 |  | 107 | 142 |  | 114 | 110 |
|  | N150 | 129 | 126 |  | 120 | 177 |  | 124 | 144 |
| Analysis of variance | |  |  |  |  |  |  |  |  |
| Biochar rate | | ns | * |  | * | ns |  | ns | ns |
| N rate | | * | * |  | * | * |  | * | * |
| Biochar rate × N rate | | ns | ns |  | ns | ns |  | ns | ns |
| Internal N use efficiency (kg kg^–1^) | | | | | | | | | |
| C0 | N90 | 50.2 | 55.2 |  | 65.0 | 49.5 |  | 50.4 | 52.7 |
|  | N150 | 49.5 | 49.5 |  | 64.5 | 51.7 |  | 53.5 | 48.2 |
| C20 | N90 | 50.8 | 58.7 |  | 65.4 | 55.3 |  | 55.0 | 57.2 |
|  | N150 | 43.2 | 56.7 |  | 69.2 | 49.5 |  | 59.4 | 50.4 |
| Analysis of variance | |  |  |  |  |  |  |  |  |
| Biochar rate | | ns | * |  | ns | ns |  | * | * |
| N rate | | ns | * |  | ns | ns |  | * | * |
| Biochar rate × N rate | | ns | ns |  | ns | ns |  | ns | ns |
| Grain yield (t ha^–1^) | | | | | | | | | |
| C0 | N90 | 5.22 | 6.62 |  | 7.93 | 7.53 |  | 5.65 | 5.69 |
|  | N150 | 5.94 | 7.08 |  | 8.45 | 8.73 |  | 7.22 | 7.09 |
| C20 | N90 | 5.33 | 6.46 |  | 7.00 | 7.85 |  | 6.27 | 6.29 |
|  | N150 | 5.57 | 7.15 |  | 8.30 | 8.77 |  | 7.36 | 7.26 |
| Analysis of variance | |  |  |  |  |  |  |  |  |
| Biochar rate | | ns | ns |  | * | ns |  | ns | * |
| N rate | | * | * |  | * | * |  | * | * |
| Biochar rate × N rate | | ns | ns |  | ns | ns |  | ns | ns |

†C0 and C20 represent 0 and 20 t biochar ha^–1^, respectively.

‡N90 and N150 represent 90 and 150 kg N ha^–1^, respectively.

* and ns denote significance and non-significance at the 0.05 probability level, respectively.

**Table S2** Analysis of variance for the effects of biochar and N rates on total NH_3_ volatilization, soil ^15^N abundance, ^15^N uptake, and soil pH, total N content, available N content and invertase activity in the late season in 2017

| Biochar rate† | N rate‡ | Total NH_3_ volatilization  (g m^–2^) | Soil ^15^N abundance  (%) | ^15^N uptake  (g m^–2^) | Soil pH | Soil total N content  (g kg^–1^) | Soil available N content  (mg kg^–1^) | Soil invertase activity  (mg g^–1^ h^–1^) |
| --- | --- | --- | --- | --- | --- | --- | --- | --- |
| C0 | N90 | 0.92 | 0.395 | 2.90 | 6.30 | 1.54 | 151 | 3.75 |
|  | N150 | 1.52 | 0.406 | 4.61 | 6.33 | 1.33 | 157 | 4.05 |
| C20 | N90 | 1.16 | 0.407 | 2.39 | 6.57 | 1.55 | 154 | 3.36 |
|  | N150 | 2.00 | 0.412 | 4.53 | 6.66 | 1.81 | 156 | 3.27 |
| Analysis of variance | |  |  |  |  |  |  |  |
| Biochar rate | | * | * | * | * | * | ns | * |
| N rate | | * | * | * | ns | ns | * | ns |
| Biochar rate × N rate | | ns | ns | ns | ns | ns | ns | ns |

†C0 and C20 represent 0 and 20 t biochar ha^–1^, respectively.

‡N90 and N150 represent 90 and 150 kg N ha^–1^, respectively.

* and ns denote significance and non-significance at the 0.05 probability level, respectively.
